# In Vivo Visualization and Quantification of Brain Heat Shock Protein 90 with [^11^C]HSP990 in Healthy Aging and Neurodegeneration

**DOI:** 10.2967/jnumed.124.268961

**Published:** 2025-06

**Authors:** Romy Cools, Koen Vermeulen, Eline Vonck, Veerle Baekelandt, Cassis Varlow, Valeria Narykina, Christopher Cawthorne, Koen Van Laere, Wim Vanduffel, Neil Vasdev, Guy Bormans

**Affiliations:** 1 Laboratory for Radiopharmaceutical Research, Department of Pharmacy and Pharmacologic Sciences, KU Leuven, Leuven, Belgium;; 2 Nuclear Medical Applications Institute, Belgian Nuclear Research Centre, Mol, Belgium;; 3 Laboratory for Neurobiology and Gene Therapy, Leuven Brain Institute, KU Leuven, Leuven, Belgium;; 4 Brain Health Imaging Centre, Centre for Addiction and Mental Health and University of Toronto, Toronto, Canada;; 5 Switch Laboratory, Department of Cellular and Molecular Medicine, VIB-KU Leuven, Leuven, Belgium;; 6 Nuclear Medicine and Molecular Imaging, Department of Imaging and Pathology, KU Leuven, Leuven, Belgium; and; 7 Laboratory for Neuro- and Psychophysiology, Department of Neuroscience, KU Leuven, Leuven, Belgium

**Keywords:** Hsp90, PET, brain, neurodegeneration

## Abstract

Heat shock protein 90 (Hsp90) is essential for maintaining cellular proteostasis and may play an important role in the development of neurodegenerative proteinopathies. Therefore, we aimed to develop an Hsp90-specific PET brain tracer to quantify Hsp90 expression in the brain in vivo in order to explore its potential as a biomarker for neurodegenerative disease characterization and to support Hsp90-targeted drug development. **Methods:** We developed the radiosynthesis of (*R*)-2-amino-7-(4-fluoro-2-(6-(methoxy-^11^C)pyridin-2-yl)phenyl)-4-methyl-7,8-dihydropyrido[4,3-*d*]pyrimidin-5(6*H*)-one, [^11^C]HSP990, and validated the tracer using in vitro autoradiography, in vitro brain homogenate saturation binding, ex vivo biodistribution, and in vivo PET imaging in rodent models of Alzheimer disease (AD) and Parkinson disease versus healthy age-matched and young controls. Human brain samples from AD patients and healthy subjects were included in our in vitro binding studies. A nonhuman primate PET brain study with arterial blood sampling was conducted under baseline and blocking conditions. **Results:** In vitro and in vivo [^11^C]HSP990 studies in rodents and a nonhuman primate revealed saturable Hsp90 binding pools in natural killer lymphocytes, bone marrow, and notably the brain, where the highest binding was observed, particularly in gray matter. Blocking studies indicated that saturable Hsp90 in natural killer lymphocytes considerably influences the pharmacokinetics of Hsp90-targeting probes, which is critical for Hsp90 drug development. In vitro [^3^H]HSP990 brain homogenate saturation binding assays suggested that the tracer binds a distinct subfraction of the total Hsp90 pool, which is significantly diminished in both rodent and human AD brain tissue compared with age-matched controls. In vivo PET imaging confirmed reduced [^11^C]HSP990 brain binding on aging and an even stronger decrease in AD mice, suggesting that Hsp90 depletion may impair protein quality control and accelerate proteinopathies. **Conclusion:** [^11^C]HSP990 is a promising Hsp90-specific tracer and reveals strong Hsp90 binding in the brain. Uniformly reduced tracer binding was observed in AD brain tissue compared with age-matched controls. [^11^C]HSP990 holds potential as a biomarker for neurodegenerative disease characterization and progression, and it may aid in patient stratification and therapy monitoring. Human [^11^C]HSP990 PET neuroimaging studies are under way to investigate whether these findings translate to humans.

## Visual Abstract

**Figure:**
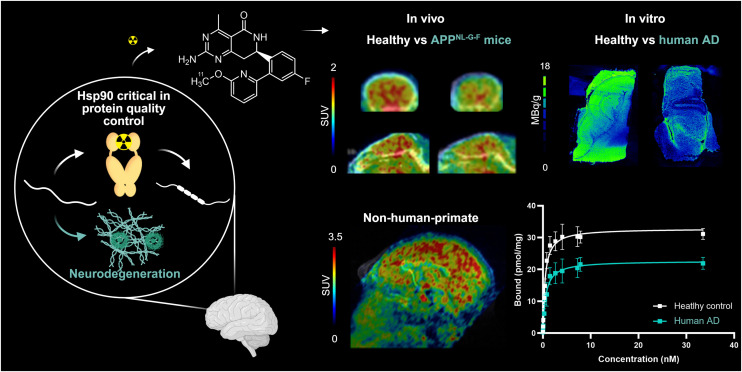

Heat shock protein 90 (Hsp90), present in 4 isoforms, is a key chaperone in the protein quality control system, maintaining cellular proteostasis by stabilizing, folding and refolding, and regulating many client proteins (1). Accordingly, Hsp90 function is implicated in diseases associated with proteotoxic stress, such as neurodegenerative disorders and cancer (2,3). The role of Hsp90 in neurodegenerative diseases, including Alzheimer disease (AD), Parkinson disease (PD), amyotrophic lateral sclerosis, and Huntington disease, remains controversial in the literature and has been linked to both protective functions and pathologic roles.

Proteins involved in aggregation, such as β-amyloid, phosphorylated tau, and α-synuclein (αSyn), are reported Hsp90 clients (4–6). Some studies have shown that upregulated Hsp90 colocalized with these aggregates in a neurodegenerative brain, potentially contributing to disease pathology by exacerbating aggregate accumulation and hindering misfolded protein degradation (2,6). This has sparked interest in Hsp90 inhibitors, to suppress its aberrant neuronal activity, as potential treatments for neurodegenerative disorders (7). Conversely, reduced Hsp90 levels have been linked to neuronal cell death. In this context, induction of the heat shock response through Hsp90 inhibition has been explored to upregulate chaperone function, thereby reducing protein aggregation and supporting cytoprotection (1,7–11). For instance, (*S*)-2-fluoro-6-((tetrahydrofuran-3-yl)amino)-4-(3,6,6-trimethyl-4-oxo-4,5,6,7-tetrahydro-1*H*-indol-1-yl)benzamide) (SNX-0723) and 9-(3-(*tert*-butylamino)propyl)-8-((6-iodobenzo[*d*][1,3]dioxol-5-yl)thio)-9*H*-purin-6-amine (PU-AD) (Fig. 1) have shown promise in preclinical studies by preventing αSyn oligomerization and rescuing striatal dopamine levels in a PD rat model and by inducing the degradation of misfolded proteins and restoring memory in an AD mouse model, respectively (2,12). PU-AD was evaluated in clinical trials for AD, amyotrophic lateral sclerosis, and glioblastoma, but these studies were withdrawn or terminated and results have yet to be published (13–15). The compound (*R*)-2-amino-7-(4-fluoro-2-(6-methoxypyridin-2-yl)phenyl)-4-methyl-7,8-dihydropyrido[4,3-*d*]pyrimidin-5(6*H*)-one (HSP990) (Fig. 1) has also shown therapeutic potential, improving cognitive function in AD mouse models and reducing huntingtin aggregation in Huntington disease, but exhibited neurotoxicity in a phase I trial for solid tumors (16–18).

**FIGURE 1.**
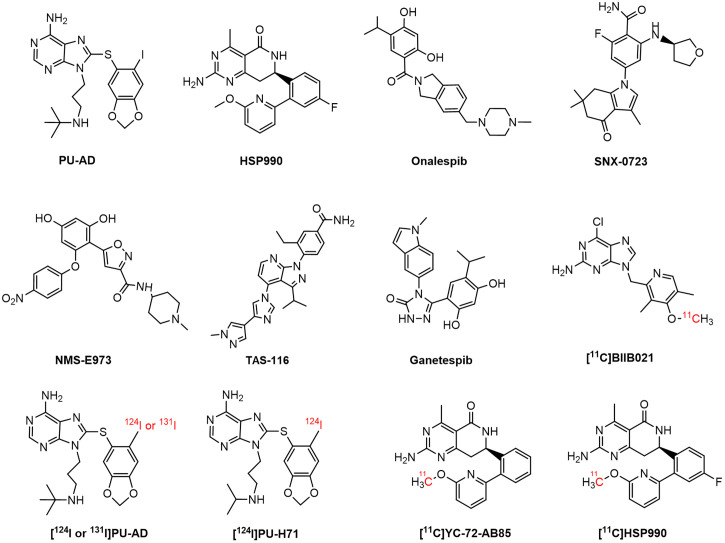
Chemical structure of Hsp90 inhibitors and PET brain tracers.

The potential clinical efficacy of Hsp90 inhibitors depends on their ability to exert therapeutic effects at safe doses. Accurate measurement of Hsp90 occupancy in the brain using PET can significantly contribute to determining optimal dosing regimens and avoiding toxic effects (19). Furthermore, Hsp90 PET brain imaging may provide insights into the role of Hsp90 in neurologic diseases.

Only a few Hsp90-targeting PET probes that can permeate the blood–brain barrier have been developed so far (2,20,21). [^124^I]PU-AD (Fig. 1) showed higher hippocampal retention in human AD brain than in controls on static delayed (3 h after injection) PET. However, absolute brain [^124^I]PU-AD concentrations were very low, and ^124^I produces low-quality PET images and exposes patients to high radiation because of its long half-life (2,22). More recently, 6-chloro-9-((4-(methoxy-^11^C)-3,5-dimethyl-2-pyridinyl)methyl]-9*H*-purin-2-amine ([^11^C]BIIB021) (Fig. 1) was developed, exhibiting Hsp90-specific binding in the rat brain but showing the presence of brain radiometabolites, which may complicate PET image quantification (21). Our group previously developed (*R*)-2-amino-7-(2-(6-(methoxy-^11^C)pyridin-2-yl)phenyl)-4-methyl-7,8-dihydropyrido[4,3-*d*]pyrimidin-5(6*H*)-one ([^11^C]YC-72-AB85) (Fig. 1), demonstrating reversible Hsp90-specific brain binding in healthy rodents and a nonhuman primate (NHP) (20), but it has not been explored in neurodegeneration. Given that HSP990 has already been used in a clinical trial, this study sought to develop and evaluate [^11^C]HSP990 (Fig. 1) as a Hsp90 PET tracer to elucidate saturable Hsp90 binding and its role in health and neurodegenerative disease and to advance clinical translation.

## MATERIALS AND METHODS

The full materials and methods are provided in the supplemental materials (Supplemental Figs. 1–7; Supplemental Tables 1 and 2 [supplemental materials are available at http://jnm.snmjournals.org]) (23–50).

### Radiosynthesis

^11^C was produced via proton irradiation of a 95% nitrogen and 5% hydrogen gas mixture in a Cyclone 18/9 cyclotron (IBA Louvain-la-Neuve) and obtained as [^11^C]CH_4_ by a ^14^N(p,α)^11^C nuclear reaction. [^11^C]CH_4_ was converted to [^11^C]CH_3_I and reacted with the pyridinol precursor and cesium carbonate in dimethylformamide at 120°C for 3 min to provide [^11^C]HSP990, which was purified by high-performance liquid chromatography.

### In Vitro Autoradiography

Frozen rodent brain sections were preincubated with vehicle, HSP990 (Biorbyt), onalespib (Selleckchem Chemicals), PU-H71 (Selleckchem Chemicals), SNX-0723 (MedChem Express), PU-AD (MedChem Express), ganetespib (Selleckchem Chemicals), or NMS-E973 (Selleckchem Chemicals). After washing, sections were incubated with [^11^C]HSP990. Frozen human hippocampal brain sections were coincubated with [^3^H]HSP990 (Novandi Chemistry AB) and vehicle, HSP990, or onalespib. After tracer incubation, sections were washed, air-dried, exposed to a phosphor storage screen, and quantified as digital light units per square millimeter or MBq/g.

### In Vitro Tissue Homogenate Binding

Rodent brain and human medial frontal lobe homogenates (100 mg/mL in a Tris and sodium chloride buffer) were diluted (5–10 µg of tissue per well) and incubated with [^3^H]HSP990 (0.01–40 nM). Nonspecific binding was determined by coincubation with HSP990. Bound tracer was separated by filtration, quantified via scintillation counting, and expressed as fmol/mg versus incubated tracer concentration in nanomolars. The maximum number of binding sites (B_max_) and dissociation constant (K_d_) values were calculated using nonlinear regression.

### In Vitro Blood Cell Binding

Freshly isolated rat red blood cells and fluorescence-activated cell–sorted T, B, and natural killer (NK) cells were incubated with [^11^C]HSP990, washed, and collected for γ-counting and cell counting. Results were expressed as the percentage of the applied radioactivity bound to 10^6^ cells.

### Ex Vivo Biodistribution

Ex vivo biodistribution studies were conducted in healthy Wistar and Sprague–Dawley rats, young C57BL/6 mice, APP^NL-G-F^ mice, and age-matched controls. Animals were injected with [^11^C]HSP990 and killed at 10, 30, or 60 min after injection, and then organs were collected for γ-counting. Pretreatment with vehicle, HSP990, or SNX-0723 was done in Wistar rats. Results were expressed as percentage injected dose and SUV.

### In Vivo PET Imaging

In vivo PET imaging was conducted on C57BL/6, APP^NL-G-F^, and AAV-αSyn PD mice; Wistar rats; and 1 NHP. Animals were injected with [^11^C]HSP990, and dynamic scans were acquired for 90 min. Pretreatment and displacement studies were performed using vehicle, onalespib, SNX-0723, PU-AD, or HSP990. In addition, AAV-αSyn PD mice were scanned with [^18^F]FE-PE2I (UZLeuven) to determine striatal nondisplaceable binding potential (BP_ND_). The NHP underwent arterial blood sampling and metabolite analysis to derive the plasma input function. PET data were reconstructed and analyzed with PMOD software (PMOD Technologies) to generate SUV time–activity curves.

## RESULTS

### Radiosynthesis of [^11^C]HSP990

[^11^C]HSP990 was synthesized by *O*-methylation of the pyridinol precursor with a radiochemical yield of 36% ± 9%, more than 99% radiochemical purity, and molar activity of 150 ± 50 GBq/µmol at the end of synthesis (*n* = 5; Supplemental Figs. 8–10).

### In Vitro Autoradiography of Healthy and Diseased Brain Tissue

**FIGURE 2.**
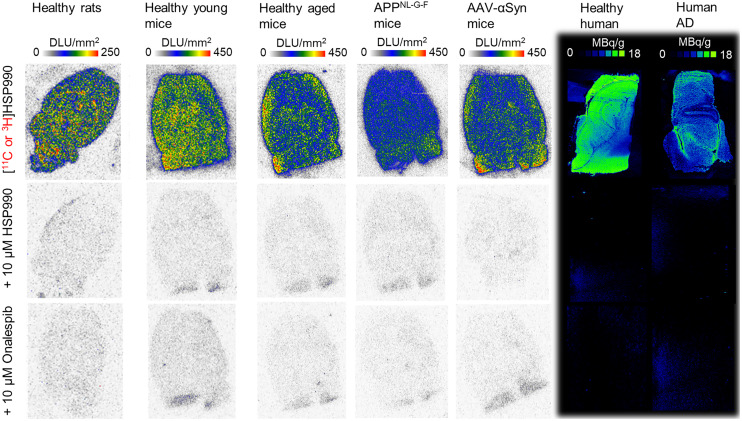
In vitro autoradiography of healthy and diseased brain tissue. DLU = digital light units.

In vitro autoradiography revealed high and consistent tracer binding to brain sections from healthy rodents and human brain tissue (Fig. 2). Preincubation with the reference compound and various nonstructurally related Hsp90 inhibitors significantly reduced tracer binding, confirming saturable and Hsp90-specific binding in both rodent and human brain tissue (Fig. 2; Supplemental Fig. 11). Tracer binding was lower in brain tissue from healthy aged mice than in that from young mice. In addition, pathologic sections from of APP^NL-G-F^ AD mice exhibited significantly reduced binding compared with age-matched controls, likely due to decreased Hsp90α/β expression (Fig. 2; Supplemental Figs. 12 and 13). Similarly, tracer binding was significantly reduced in hippocampal sections from AD patients, particularly in gray matter regions expressing tau pathology (Fig. 2; Supplemental Figs. 12 and 14). In AAV-αSyn PD mice, no differences in [^11^C]HSP990 binding were observed between ipsilateral and contralateral brain sides or in Hsp90α/β expression, despite confirmed dopaminergic neuronal cell death (40%) and evident pSer129-αSyn pathology in affected regions (Fig. 2; Supplemental Figs. 12 and 15).

### In Vitro Brain Tissue Homogenate Saturation Binding to Healthy and AD Brain

**FIGURE 3.**
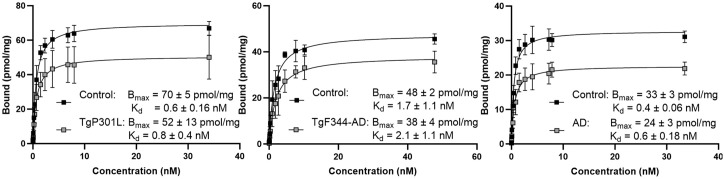
In vitro saturation binding to brain homogenate of TgP301 L mice (left), TgF344-AD rats (middle), and human AD subjects (right) vs. age-matched controls. Data are presented as mean ± SD (*n* = 2–6 per experimental group). K_d_ = dissociation constant.

Saturation binding assays using [^3^H]HSP990 were conducted on brain tissue homogenates from rodent AD models, including TgP301 L mice and TgF344 rats, as well as on the medial frontal lobe tissue of human AD patients and age-matched controls. Saturation curves demonstrated high affinity for brain Hsp90, with dissociation constants in the nanomolar range. The B_max_ values were significantly lower in AD brain tissue than in control tissue, suggesting reduced Hsp90 expression in AD, whereas the affinity of the tracer for Hsp90 remained unchanged between health and disease. BP was high and significantly different between AD and healthy brain, suggesting the tracer’s potential for imaging altered Hsp90 expression in AD patients (Fig. 3; Supplemental Table 3).

### Ex Vivo Biodistribution and Quantification of Radiometabolites in Healthy Rodents

**FIGURE 4.**
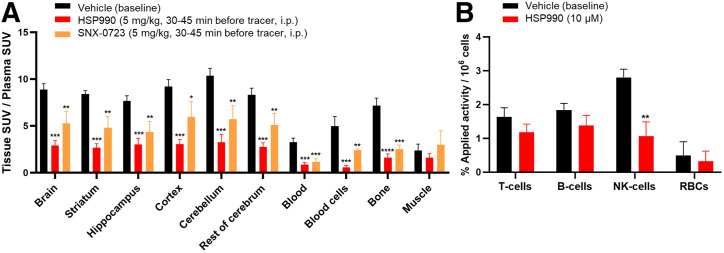
(A) Ex vivo biodistribution study under baseline and pretreatment conditions in healthy rats. (B) In vitro fluorescence-activated cell–sorted rat blood cell binding. Data are presented as mean ± SD (*n* = 3 per pretreatment). *P ≤ 0.05. **P ≤ 0.01. ***P ≤ 0.001. ****P ≤ 0.0001. i.p. = intraperitoneally; RBCs = red blood cells.

A plasma radiometabolite study in rats showed 73% ± 3% of intact tracer at 30 min after injection, with less than 1% of radiometabolites crossing the blood–brain barrier (Supplemental Table 4). Ex vivo biodistribution in healthy rodents revealed predominant hepatobiliary clearance, bone marrow accumulation, and sustained high blood SUV, suggesting slow clearance because of blood cell binding (Supplemental Fig. 16; Supplemental Table 5). Brain uptake of [^11^C]HSP990 was high, was homogeneous across regions, and persisted over time (Supplemental Fig. 16; Supplemental Table 6). Pretreatment with HSP990 or SNX-0723 in rats blocked tracer binding in blood cells, increasing the free tracer in plasma available to bind brain Hsp90 (Supplemental Table 6). Therefore, organ-to-plasma SUV ratios were evaluated. These were significantly decreased in all brain regions, blood cells, blood-rich organs, and bone marrow, indicating saturable and Hsp90-specific tracer binding (Fig. 4).

### In Vitro Blood Binding

In vitro rodent blood binding studies confirmed saturable Hsp90 tracer binding in blood cells, with reduced binding on HSP990 preincubation, consistent with ex vivo results. In plasma, the free tracer fraction was 10%, with a 5% increase under blocking conditions, suggesting that this portion of the protein-bound tracer is Hsp90-specific (Supplemental Table 7).

Fluorescence-activated cell–sorted blood cell studies indicated that [^11^C]HSP990 mainly accumulated in NK cells, with significant reduction in binding after HSP990 preincubation, pointing to mononuclear NK cells as the main contributors to saturable Hsp90 binding observed in blood cells in vivo (Fig. 4; Supplemental Fig. 17).

### In Vivo Brain PET in Healthy Rodents

**FIGURE 5.**
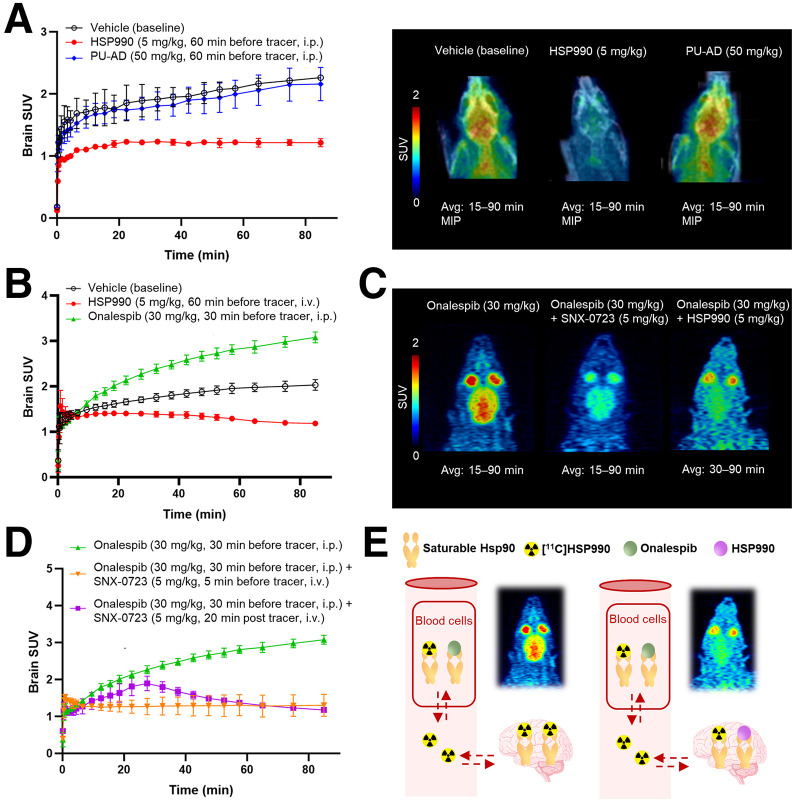
(A) In vivo PET study under baseline and pretreatment conditions in healthy mice, showing brain SUV time–activity curves (left) and images (right). (B) In vivo PET study under baseline and pretreatment conditions in healthy rats, showing brain SUV time–activity curves. (C and D) In vivo PET study under combined pretreatment and displacement conditions in healthy rats, showing images (C) and brain SUV time–activity curves (D). (E) Illustration of presumed saturable Hsp90 binding. Data are presented as mean ± SD (*n* = 3 per pretreatment). Avg = average; i.p. = intraperitoneally; i.v. = intravenously; MIP = maximum-intensity projection.

In vivo PET imaging in healthy rodents demonstrated high and sustained [^11^C]HSP990 brain uptake at baseline that decreased after HSP990 pretreatment, indicating saturable Hsp90 binding (Fig. 5A). Brain uptake remained reduced when rats were pretreated with HSP990 5 h before tracer administration, suggesting slow HSP990 brain kinetics and extended Hsp90 occupancy (Supplemental Fig. 18). In contrast, PU-AD pretreatment minimally affected brain uptake (Fig. 5A). Pretreatment with brain-impermeable onalespib increased brain uptake 2-fold because of the blocking effect in peripheral blood cells (Figs. 5B–5E). Combining onalespib with SNX-0723 decreased brain uptake below the control time–activity curve, confirming in vivo Hsp90-specific binding (Figs. 5B–5E). Pretreatment with onalespib followed by tracer injection and subsequent HSP990 administration resulted in fast dissociation of [^11^C]HSP990 from brain Hsp90, showing that binding is reversible. Therefore, brain kinetics appear slow because of sustained tracer delivery resulting from slow tracer dissociation from the peripheral blood cell compartment (Figs. 5B–5E).

### In Vivo Brain PET in AD and PD Rodent Models

**FIGURE 6.**
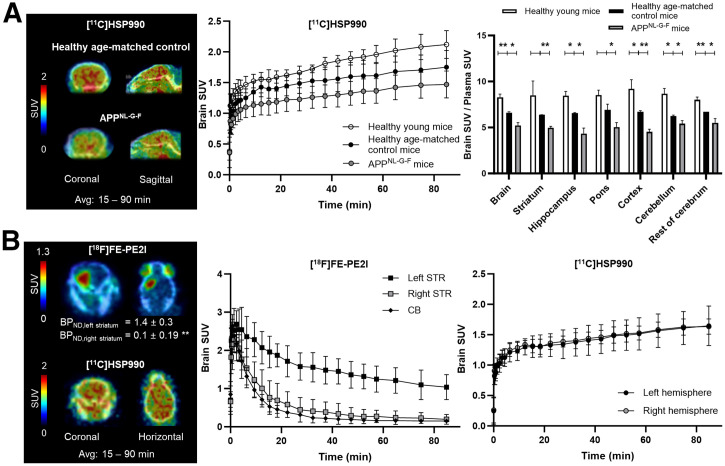
(A) In vivo [^11^C]HSP990 PET imaging with corresponding brain SUV time–activity curves and ex vivo biodistribution, showing brain-to-plasma SUV in APP^NL-G-F^ mice, healthy age-matched control mice, and healthy young mice. Data are presented as mean ± SD (*n* = 3–5 per experimental group). (B) In vivo [^18^F]FE-PE2I and [^11^C]HSP990 PET imaging in AAV-αSyn mice with corresponding brain SUV time–activity curves. Data are presented as mean ± SD (*n* = 4). *P ≤ 0.05. **P ≤ 0.01. Avg = average; CB = cerebellum; STR = striatum.

In vivo PET imaging showed reduced [^11^C]HSP990 brain uptake in APP^NL-G-F^ mice compared with age-matched controls, particularly in the striatum, hippocampus, and cortex, as confirmed by ex vivo biodistribution (Fig. 6A; Supplemental Fig. 19). Healthy aged mice also exhibited lower brain tracer uptake than that of young mice, along with reduced blood cell binding, affecting plasma tracer delivery to brain (Supplemental Fig. 19; Supplemental Table 8). Therefore, brain-to-plasma SUV ratios more reliably represent brain uptake, which were significantly reduced in APP^NL-G-F^ mice versus aged-matched controls and in healthy aged versus young mice in almost all brain regions (Fig. 6A).

In AAV-αSyn PD mice, brain uptake of [^11^C]HSP990 was homogeneous, showing no differences between ipsilateral and contralateral hemispheres (Fig. 6B; Supplemental Fig. 20). In contrast, [^18^F]FE-PE2I scans demonstrated lower retention in the ipsilateral pathologic striatum (right) than in the contralateral striatum (left), resulting in significantly lower BP_ND_ of [^18^F]FE-PE2I in the right versus left striatum and confirming robust αSyn-induced presynaptic dopaminergic neurodegeneration in the affected hemisphere (Fig. 6B).

### In Vivo Brain PET in NHP

**FIGURE 7.**
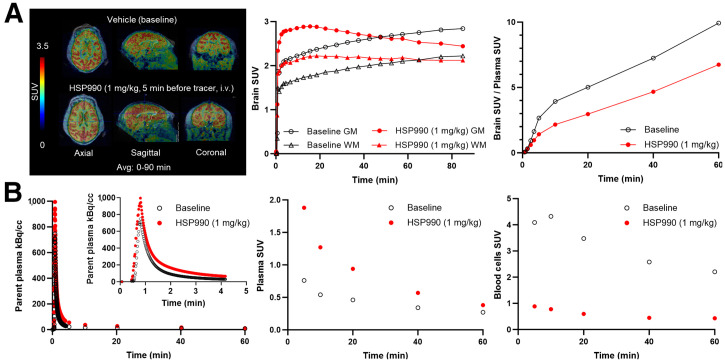
(A) In vivo PET brain imaging under baseline and pretreatment conditions in NHP with corresponding brain SUV time–activity curves and brain-to-plasma SUV time–activity curves. (B) Arterial blood–derived, radiometabolite-corrected plasma input curve with plasma SUV and blood cell SUV. Avg = average; GM = gray matter; i.v. = intravenously; WM = white matter.

Dynamic baseline PET scans in the NHP demonstrated high and sustained [^11^C]HSP990 brain uptake across all cerebral and cerebellar regions, with higher accumulation in gray matter than in white matter (Fig. 7A; Supplemental Fig. 21). At 40 min after injection, more than 85% of the tracer in plasma remained intact and 90% of the radioactivity in the blood was associated with blood cells. Low-dose HSP990 pretreatment significantly reduced activity in blood cells, resulting in increased plasma SUV because of tracer displacement from Hsp90 binding sites (Fig. 7B; Supplemental Table 9). Enhanced delivery of unbound tracer from plasma contributed to higher initial brain activity values in blocking conditions than at baseline and apparently slow brain tracer kinetics. Brain-to-plasma ratios were higher at baseline than in blocking conditions across all brain regions, indicating specific binding in the NHP brain and the absence of a visually demonstrable, suitable reference region devoid of specific binding for analysis (Fig. 7A).

## DISCUSSION

Hsp90 is critical in the protein quality control system for regulating the folding, maturation, and clearance of more than 400 client proteins, ensuring cellular proteostasis (1). In conditions such as neurodegenerative diseases and cancer, cells increasingly rely on Hsp90, sparking interest in Hsp90-targeted therapies. Despite promising preclinical results, many Hsp90 inhibitors failed in clinical trials, particularly in oncology (10), because of poor pharmacokinetic profiles, lack of specificity, resistance, and dose-limiting toxicities (51). Recently, the first Hsp90 inhibitor, 3-ethyl-4-[3-(1-methylethyl)-4-[4-(1-methyl-1*H*-pyrazol-4-yl)-1*H*-imidazol-1-yl]-1*H*-pyrazolo[3,4-*b*]pyridin-1-yl]-benzamide (TAS-116), was approved for gastrointestinal tumors in Japan (52). However, the development of Hsp90-targeting drugs with a robust therapeutic index in the central nervous system context remains challenging. Therefore, Hsp90 brain PET could advance the field by enabling drug-occupancy studies and providing in vivo insights into Hsp90’s role in disease.

This study aimed to expand the limited library of Hsp90 PET brain tracers by evaluating [^11^C]HSP990 as an Hsp90 PET tracer. The tracer showed high specificity for Hsp90, validated through in vitro autoradiography, ex vivo biodistribution, and in vivo PET blocking studies. [^11^C]HSP990 demonstrated a pharmacokinetic profile comparable to that of our previous probe, [^11^C]YC-72-AB85, with slow metabolism and hepatobiliary clearance. Its brain uptake exceeded that of the earlier Hsp90 PET tracers [^11^C]YC-72-AB85, [^124^I]PU-AD, and [^11^C]BIIB021 (2,20,21). Biodistribution pretreatment studies confirmed saturable Hsp90 binding in the brain, blood cells, blood-rich organs, and bone marrow, as previously observed for [^11^C]YC-72-AB85 (20). Of all inhibitors used, PU-AD unexpectedly did not reduce in vivo [^11^C]HSP990 binding in healthy mouse brain. Although PU-AD was reported to dissociate faster from healthy Hsp90 than from the epichaperome–Hsp90 complex found in disease conditions, its dissociation half-life for normal Hsp90 is still 2 h, so we anticipated occupancy of brain Hsp90 (53).

The saturable tracer binding in blood cells was further investigated with [^11^C]HSP990, revealing significant accumulation in peripheral blood mononuclear cells, especially NK cells, which influences the pharmacokinetics of Hsp90-targeted probes. Dose-dependent nonlinear kinetics has been reported for Hsp90 inhibitors (54). Badolo et al. (54) found no significant difference in plasma and the blood-free fraction after in vitro incubation, possibly because of saturation of the blood cell binding capacity at a 1 µM concentration. Our ex vivo monkey blood analysis also showed a marked reduction in [^11^C]HSP990 binding, indicating blood cell saturation after pretreatment with a low dose (1 mg/kg) of HSP990. These findings have important implications for dosing optimization in Hsp90-targeted therapy to mitigate toxicity issues, such as upcoming microdosing studies (17), considering the possible inhibitory effects on NK cells (55) and altered pharmacokinetics of Hsp90-targeted therapeutics. This underscores the value of Hsp90 PET for monitoring in vivo Hsp90 occupancy in the blood and targeted tissues.

The high yet saturable in vivo [^11^C]HSP990 brain binding is intriguing. The brain has the highest uptake compared with other organs, which is consistent with the findings of Barrott et al. (56) and Maiti and Picard (8), despite earlier conflicting reports (3). In vitro [^3^H]HSP990 saturation binding assays yielded high in vitro BP values (∼7000), which are likely an overestimation of in vivo BP, possibly because of relatively low levels of free radioligand available in the brain in vivo, as observed with several other brain tracers (57). We obtained an in vitro B_max_ of 33 ± 3 pmol/mg protein in healthy human brain tissue, representing 0.3% of total brain cellular protein mass, which is 3 times lower than reported brain Hsp90 levels (58). This suggests [^3^H]HSP990 binds to an active subpool of Hsp90, comprising approximately 30% of total brain Hsp90, and supports the hypothesis by Bhattacharya et al. (59) of distinct Hsp90 pools, 1 functionally latent and the other active and critical for survival thresholds.

To explore whether saturable Hsp90 expression is affected in neurodegenerative brain disorders, saturation binding assays were explored on AD brain tissue and revealed reduced B_max_ compared with that of age-matched controls. However, the affinity remained unchanged, contrasting the reported high-affinity epichaperome–Hsp90 complex observed in AD-affected brain using [^124^]PU-AD (53). This resulted in significantly lower BP values for [^3^H]HSP990 in AD tissue, suggesting the potential to detect diminished Hsp90 levels in AD-diseased versus healthy brain using [^11^C]HSP990 PET.

Our in vitro autoradiography and in vivo brain distribution studies using [^11^C]HSP990 showed reduced saturable Hsp90 tracer binding in rodent AD brain compared with age-matched controls, particularly in key AD-affected regions, including hippocampus and neocortex. In vitro [^3^H]HSP990 autoradiography of hippocampal human AD brain tissue confirmed lower tracer binding in regions with phosphorylated tau accumulation, consistent with studies illustrating an inverse relationship between aggregated tau and Hsp90 expression in AD brain (60). However, this contrasts [^131^I]PU-AD autoradiography, which showed increased uptake attributed to epichaperome binding in hippocampal regions colocalizing with tau fibrils in PS19 AD mice (2). In addition, the observed decrease in [^11^C]HSP990 brain uptake with aging in healthy mice mirrors findings from proteomic studies (9,61). Bhattacharya et al. (59) reported that the brain is particularly sensitive to reductions in Hsp90, requiring at least 75% of normal levels to avoid proteostatic collapse. The age-related decrease and substantial reduction in AD brain detected in vivo using [^11^C]HSP990 PET may suggest that depletion of (active) Hsp90 reduces the capacity of the protein quality control system, impairing its ability to prevent protein aggregation and potentially accelerating the onset of neurodegenerative diseases. These results indicate that [^11^C]HSP990 PET could serve as a biomarker for assessing changes in Hsp90 expression, offering a tool for studying early disease progression and therapeutic efficacy. Furthermore, this work supports the hypothesis that enhancement of Hsp90 levels through Hsp90 inducers (8,9), positive allosteric modulators of Hsp90 (7,10), or Hsp90 inhibitors that activate the heat shock response (7) could be a therapeutic strategy for conditions characterized by protein aggregation, such as AD and PD.

However, our [^11^C]HSP990 studies in PD mice did not reveal detectable differences in tracer brain binding to the αSyn-affected versus control hemisphere. This may be due to the advanced stage of pathology in the animals at the time of scanning, with a more than 90% reduction in nigrostriatal dopaminergic projections as measured by dopamine transporter PET. Therefore, clinical [^11^C]HSP990 PET in PD patients could provide more insights into the potential role of Hsp90 in synucleinopathies.

Finally, [^11^C]HSP990 was evaluated in NHP PET studies with arterial sampling to provide kinetic estimates for first-in-human studies. As in rodents, saturable Hsp90 binding was observed throughout the brain, with higher uptake in gray matter. However, the slow brain tracer kinetics due to peripheral blood cell binding may complicate straightforward quantification of Hsp90 binding and drug occupancy and require extended scan durations in human [^11^C]HSP990 brain studies.

## CONCLUSION

[^11^C]HSP990 is a promising Hsp90 PET imaging probe that revealed saturable Hsp90 binding pools in white blood cells, blood-rich organs, bone marrow, and notably the brain, where the highest Hsp90-specific binding was observed. The presence of saturable Hsp90 binding in NK cells has significant implications for Hsp90 drug development and highlights the value of [^11^C]HSP990 PET in monitoring Hsp90 occupancy to optimize dosing regimens for next-generation Hsp90 therapeutics. Moreover, our findings suggest that [^11^C]HSP990 allows in vivo imaging and quantification of the active Hsp90 subpool, critical for maintaining proteostasis, and that this is significantly reduced with aging and particularly in AD brain compared with age-matched controls. This indicates its potential as a biomarker for neurodegenerative disease characterization, progression, and therapy monitoring. However, the slow brain kinetics of [^11^C]HSP990 will complicate accurate quantification of Hsp90 binding. Therefore, ongoing clinical [^11^C]HSP990 neuroimaging studies aim to further explore the potential of [^11^C]HSP990 PET to image and quantify Hsp90 in vivo.

## DISCLOSURE

This work was supported by FWO (G0A6222N, AKUL15-30/G0H1216N, and I000321N) and KU Leuven (C1, C14/21/111; C2, C24E/19/070). No other potential conflict of interest relevant to this article was reported.

## Notes

KEY POINTS **QUESTION:** Is [^11^C]HSP990 PET suitable for quantifying Hsp90 expression in healthy aging and neurodegeneration? **PERTINENT FINDINGS:** [^11^C]HSP990 is a promising Hsp90-specific brain tracer that demonstrated the highest specific binding in the brain among all studied organs and tissues. Moreover, [^11^C]HSP990 can quantify reduced Hsp90 levels in AD brain compared with age-matched controls. **IMPLICATIONS FOR PATIENT CARE:** [^11^C]HSP990 PET holds potential for neurodegenerative disease characterization, disease monitoring, patient stratification, and therapy monitoring. Ongoing clinical [^11^C]HSP990 neuroimaging studies aim to validate these hypotheses.
